# Microstructural abnormalities in multiple system atrophy as revealed by conventional and rotating frame relaxation MRI parameters

**DOI:** 10.1038/s41598-025-10812-6

**Published:** 2025-07-24

**Authors:** Antonietta Canna, Silvia Mangia, Shalom Michaeli, Sara Ponticorvo, Maria Claudia Russillo, Valentina Andreozzi, Renzo Manara, Francesco Di Salle, Marina Picillo, Paolo Barone, Fabrizio Esposito, Maria Teresa Pellecchia

**Affiliations:** 1 Montreal Neurological Institute and Hospital, the Neuro, McGill University, Montreal, QC Canada; 2 Center for Magnetic Resonance Research (CMRR), Department of Radiology, University of Minnesota, Minneapolis (MN), USA; 3 Neuroradiology Unit, University of Padua, Padua, Italy; 4 Department of Medicine, Surgery and Dentistry Scuola Medica Salernitana, Section of Neurosciences, University of Salerno, Salerno, Italy; 5 IRCCS SYNLAB SDN, Naples, Italy; 6 Department of Advanced Medical and Surgical Sciences, University of Campania Luigi Vanvitelli, Naples, Italy

**Keywords:** Adiabatic T1ρ, T2ρ, MT, R1, R2*, MSA, Rotating frame relaxation (RFR), Biomarkers, Neurology

## Abstract

Rotating frame relaxation (RFR) techniques provide robust and sensitive quantitative MRI (qMRI) metrics able to detect subtle alterations of brain tissue integrity in multiple neurological disorders. We performed a cross-sectional qMRI study in 16 patients with Multiple System Atrophy (MSA) and 14 age- and sex-matched healthy controls. The MRI protocol included the acquisition of RFR metrics, namely adiabatic T1ρ and T2ρ relaxation time constants, along with the acquisition of conventional relaxation metrics, namely R1 and R2*, and a semi-quantitative metric describing magnetization transfer (MT). Between-group comparisons of voxel-based and region-based outcomes were obtained both with and without accounting for the effect of atrophy. Significant brain atrophy was observed in the MSA patients in the white matter and grey matter of the cerebellum, and frontal white matter. On the other hand, group differences of virtually all relaxation metrics were observed for both voxel-wise and ROI analyses in many brain areas. These differences were reduced in spatial extent and/or effect size when the atrophy effect was accounted for. However, T1ρ values remained significantly longer in patients’ ROIs encompassing both cerebellar grey matter and white matter, while MT, and R1 values were smaller. Cerebellar gray matter ROIs of patients also exhibited longer T2ρ and smaller R2*. Other voxel-wise group differences were still present after atrophy correction in several clusters, including among others, longer T1ρ in patients’ putamen, and longer T1ρ and T2ρ and smaller MT in the patients’ pons. Altogether, our findings suggest multiple processes of tissue integrity loss during the course of neurodegeneration that go beyond macrostructural alterations. We conclude that adiabatic T1ρ and T2ρ are valuable MRI metrics that expand the set of multi-parametric qMRI available to characterize the brain of MSA, providing additional and specific information on the brain tissue alterations occurring in this disease.

**Supplementary Information:**

The online version contains supplementary material available at 10.1038/s41598-025-10812-6.

## Introduction

Multiple system atrophy (MSA) is a rare neurodegenerative disease characterized by neuronal loss and gliosis in multiple areas of the central nervous system^1^. MSA has two known variants, the cerebellar (MSA-C) and the putaminal (MSA-P) types, and this distinction is dependent on the distribution of pathology within the basal ganglia and the olivopontocerebellar system^2^. While structural and functional MRI have been previously applied to the study of MSA patients, it is the introduction of quantitative MRI (qMRI) measures that has shown potential to improve the diagnostic accuracy of the pathology^3^. Indeed, the acquisition of qMRI measures like the longitudinal relaxation rate constant R1 (≡ 1/T1) or transverse relaxation rate constant R2 (≡ 1/T2) maps improves the characterization of brain tissue by enhancing the image contrast and providing quantitative relationships between the MRI signal changes and the tissue alterations as could be revealed by histochemistry and histology^4^. These measures, often combined with that of the magnetization transfer (MT) effect, have been previously used to assess tissue-specific alterations in neurodegenerative diseases^5^.

Rotating frame relaxation (RFR) metrics, including longitudinal (T1ρ) and transverse (T2ρ) relaxation time constants, are being increasingly exploited to characterize tissue integrity in clinical pathological models as complementary biomarkers of phenomena occurring at cellular^6^ or molecular scales, since these parameters detect slow molecular motional processes that occur in the order of millisecond-microsecond time scales with greater sensitivity than conventional T1 and T2 relaxations at high magnetic fields (3 Tesla and above). In addition, T2ρ provides important insights into the diffusion and exchange processes of water protons in microenvironments with different local susceptibilities^7^, reflecting iron content with higher sensitivity than conventional T2^8,9^. Alterations in RFR adiabatic T1ρ and T2ρ have been reported in Parkinson’s disease^10–12^, Multiple Sclerosis^13,14^, amyotrophic lateral sclerosis (ALS)^15^ and aging^16^, but, to the best of our knowledge, RFR metrics have never been applied in MSA patients.

The goal of the current study was to exploit RFR metrics to characterize the tissue alterations in the brain of patients with MSA in comparison to that of healthy controls. In addition, the extent to which alterations of both conventional relaxation and RFR metrics are affected by the presence of atrophy has been rarely evaluated in the current literature even though the presence of neuronal loss may hide other critical brain abnormalities co-occurring with atrophy. For this reason, we performed voxel- and region-based analyses of free-precession relaxations and rotating frame relaxations, with and without accounting for the confounding effect of the tissue volume, as estimated respectively via the classical voxel-based morphometry (VBM) or volume segmentation of T1-weighted images across specific regions of interest like the cerebellum and the putamen, known to be target regions of the disease^17^. We thus designed a cross-sectional qMRI study, where MT, R1, and R2* mapping were acquired according to the well-established multi-parametric mapping (MPM) qMRI protocol^18,19^, while the RFR mapping included adiabatic T1ρ and T2ρ acquired as previously described^9,10,13,16,20^.

## Materials and methods

A group of 16 MSA patients, consecutively seen at our Movement Disorder outpatient clinic, and a group of 14 healthy control (HC) subjects were enrolled in this study. Eleven patients were classified as cerebellar (MSA-C) and 5 as parkinsonian MSA (MSA-P) according to their predominant motor presentation. Disease severity in MSA patients was evaluated using the Unified MSA Rating Scale (UMSARS) Part I (Historical Review), Part II (Motor Examination), and Part IV (Global Disability Scale)^21^. Demographic and clinical characteristics of these two groups are reported in Table 1.

MRI data were acquired on a 3 T MRI scanner, and the imaging protocol consisted of a 3D anatomical T1-weighted Magnetization Prepared RApid Gradient Echo (MPRAGE) sequence, three multi-echo 3D FLASH sequences with a voxel size equal to 1.0 × 1.0 × 1.0 (MPM protocol) and adiabatic T1ρ and T2ρ measurements (voxel size = 1.6 × 1.6 × 3.6 mm^3^). Details of the acquired sequences are reported in the Supplementary materials (see **MRI data acquisition**). The study was approved by the local Ethics committee (Comitato Etico Campania Sud) and all participants signed informed consent. The study was conducted in accordance with the Declaration of Helsinki principles.

**Table 1:** Demographic and clinical characterization of HC and MSA people.

Group	Age	F/M	Disease duration	UMSARS I	UMSARS II	UMSARS IV
*HC*	61 ± 8	10/4	–	–	–	–
*MSA patients*	64 ± 7	11/5	5 ± 1	27 ± 5	30 ± 7	2 ± 1

A total of 5 parametric maps were extracted for each subject. In particular, the MPM datasets were processed in MATLAB R2016a (The MathWorks Inc., Natick, MA, USA, https://it.mathworks.com/), using SPM12 (www.fil.ion.ucl.ac.uk/spm) and hMRI toolboxes^22^ (https://www.cbs.mpg.de/departments/neurophysics/software/hmri-toolbox) (see Supplementary Material for more details, sec. **Preprocessing of MPM maps**), to generate MT, R1 and R2* maps. The adiabatic T1ρ and T2ρ maps were obtained using a custom script written in Python v. 3.8 implementing a 2-parameter non-linear mono-exponential fitting procedure applied to the motion-corrected data, for which the MCFLIRT command of the FSL suite (https://fsl.fmrib.ox.ac.uk/fsl/) was used.

High-contrast T1w images from the MPRAGE series were used to assess the differences in grey matter (GM) and white matter (WM) volumes between the two populations, to normalize the obtained relaxometry maps in the MNI space (for the voxel-wise analysis) and to extract the masks for the regions-of-interest (ROI)-based analysis. Specific ROIs were chosen for the ROI-based analysis based on their known involvement in the pathological condition of interest^23^. In particular, the selected ROIs included left and right cerebellar WM, left and right cerebellar GM, right and left putamen, and pons. The rationale for performing both ROI and voxel-wise analyses is grounded on the recognition that the two approaches inherently lead to complimentary sensitivities, with the former being more suitable to detect subtle, yet widespread, effects, and the latter being more sensitive to well localized effects.

For creating the masks used in the voxel-wise analysis, T1-weighted (T1w) images were segmented into GM, WM, and cerebrospinal fluid (CSF), then normalized to MNI space using the DARTEL method. To analyze tissue volume and atrophy differences between healthy controls and MSA patients, GM and WM maps were modulated with Jacobian determinants and smoothed with a 6 mm Gaussian kernel. All maps were resampled to 2 × 2 × 2 mm³, and total intracranial volume was estimated. To enable differential smoothing in GM and WM, binary group masks were created by thresholding individual tissue probability maps. Only voxels consistently classified as GM or WM across all subjects were retained. A “coverage mask” was also created to ensure analysis was limited to regions common across all image types. Final smoothing was done using the hMRI toolbox with 6 mm FWHM.

For the creation of the masks used in the regions of interest (ROIs) analyses, T1w images were processed with FreeSurfer (https://surfer.nmr.mgh.harvard.edu) to extract predefined ROIs in the cerebellar GM/WM, putamen, and pons. Data from multi-echo flash and adiabatic T1ρ/T2ρ acquisitions were coregistered to the high-contrast T1w images using the FLIRT command of FSL. The transformation matrices were then inverted and applied to the ROI using the nearest neighbor interpolation in such a way to have the mean values being extracted in the native space of the considered maps. To account for varying fields of view across subjects, the ROIs were masked with individual coverage masks brought into native space. More details of the procedure to obtain the masks in normalized and native space are reported in the Supplementary Materials (sec. **Creation of masks for the voxel-wise analysis and Creation of masks for ROIs analysis**).

To detect voxel-wise differences in terms of tissue volume between HC and MSA patients, a full-factorial design was specified in SPM12 considering the group separation as a between-group effect and adding age, gender, and total intracranial volume as covariates. Statistical t-maps were obtained for the contrasts HC > MSA and HC < MSA and thresholds were applied to either *p* < 0.05 family-wise error (FWE) corrected for multiple comparisons or, for mere descriptive purposes, *p* < 0.001 (uncorrected).

To detect voxel-wise differences, separately in GM and WM, for all the considered quantitative and semi-quantitative parameters, two linear mixed effect (LME) models for each metric were fitted in MATLAB with the *fitlme* function across all voxels belonging to the group GM and WM masks. The first model included age, gender, tissue-specific partial volume (from either GM or WM) and group:

1 $$y{\text{ }}\sim {\text{ }}age{\text{ }} + {\text{ }}gender{\text{ }} + {\text{ }}group{\text{ }} + {\text{ }}volume{\text{ }} + 1|Subjects~~~~~~~$$

The second model did not include the tissue-specific partial volume:

2 $$y{\text{ }}\sim {\text{ }}age{\text{ }} + {\text{ }}gender{\text{ }} + {\text{ }}group{\text{ }} + 1|Subjects$$

Starting from the model coefficients determined with each model, F-maps were calculated for the main effects of the group factor and a combined voxel- and cluster-level threshold was applied to display statistically significant group effects. More specifically, a voxel-level threshold was set to *p* < 0.05 false discovery rate (FDR) corrected at peak level (correction implemented using DPABI software, https://rfmri.org/DPABI) and only clusters with extension higher than 30 voxels were considered.

The same LME models were also used for the ROI analysis, in which case the subject-specific metrics were first averaged across all voxels of each ROI’s mask. In the two models, while for the voxel-based analyses the tissue-specific partial volume effect was assessed from the GM or WM probability maps (as derived from the SPM segmentation algorithm), for the ROI analyses the tissue-specific partial volume effect was computed as the number of GM or WM voxels contained in each ROI in the native space of the subject (as derived from the FreeSurfer standard subject-specific reconstruction pipeline). For the ROI-based analyses, F-values were considered significant if the corresponding p-values were below 0.05 after FDR correction. Moreover, only for displaying purposes, we regressed out from the data distributions the fixed effects of either model 1 (age, gender and volume tissue) or model 2 (age and gender) variables in order to highlight the direction of the group differences detected by the two LME models.

Lastly, separately for each ROI, Spearman correlations were computed between each of 5 clinical variables (i.e., disease duration, UMSARS I, II, IV, and total UMSARS obtained as the sum of UMSARS I and II) and each of the 5 MRI metrics, the latter ones being either regressed by age and gender, or by age, gender and tissue volume. Given the exploratory nature of the correlation analyses, no correction for multiple comparison was applied.

## Results

**Fig. 1:**
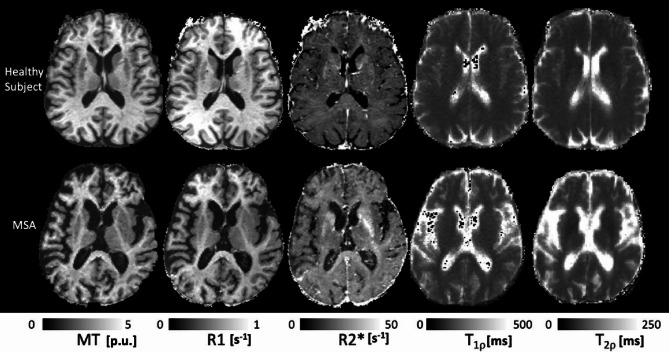
Maps of MT, R1, R2*, adiabatic T1ρ and T2ρ from one axial brain slice of a representative HC and a MSA patient. MT saturation maps are expressed in percent units [p.u.] according to the definition provided in^18^.

All enrolled participants completed the MRI protocol, provided data of sufficient quality, and were included in the statistical analyses. Representative qMRI maps are shown in Fig. 1.

### Tissue volumes

**Fig. 2:**
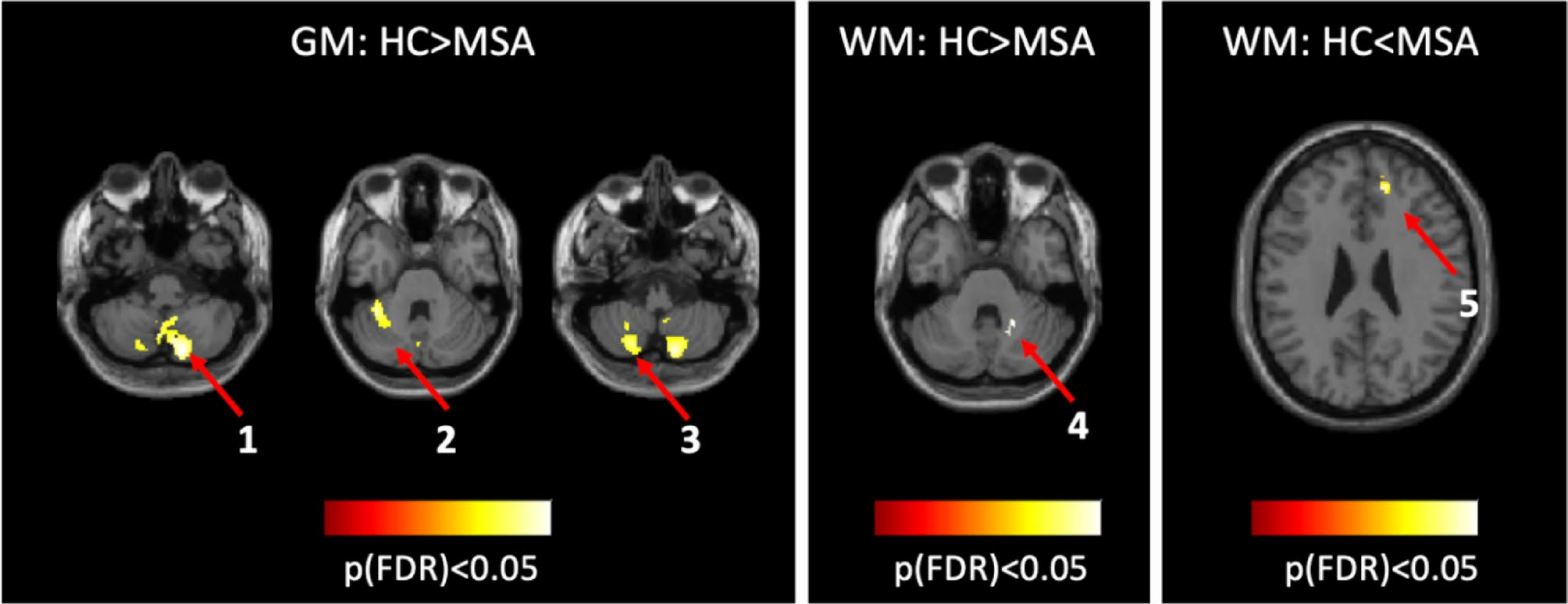
Differences in GM and WM for the comparisons between HC and MSA. Clusters Coordinates: 1= (14, −80, −44), 2= (−36, −44, −30), 3= (−22, −68, −48),4 = (22, −52, −32), 5= (14, 42, 26). Clusters 1 to 4 belong to cerebellum, while cluster 5 belongs to frontal WM.

No group differences were detected for tissue volumes at a voxel statistical threshold of *p* < 0.05 family wise (FWE) corrected. At a slightly lower threshold (*p* < 0.001 uncorrected at voxel level but corrected at the cluster level), differences emerged in the cerebellar WM and GM, and the frontal WM. These results remained significant after FDR corrections (Fig. 2).

### Voxel-wise analysis in grey matter

**Fig. 3:**
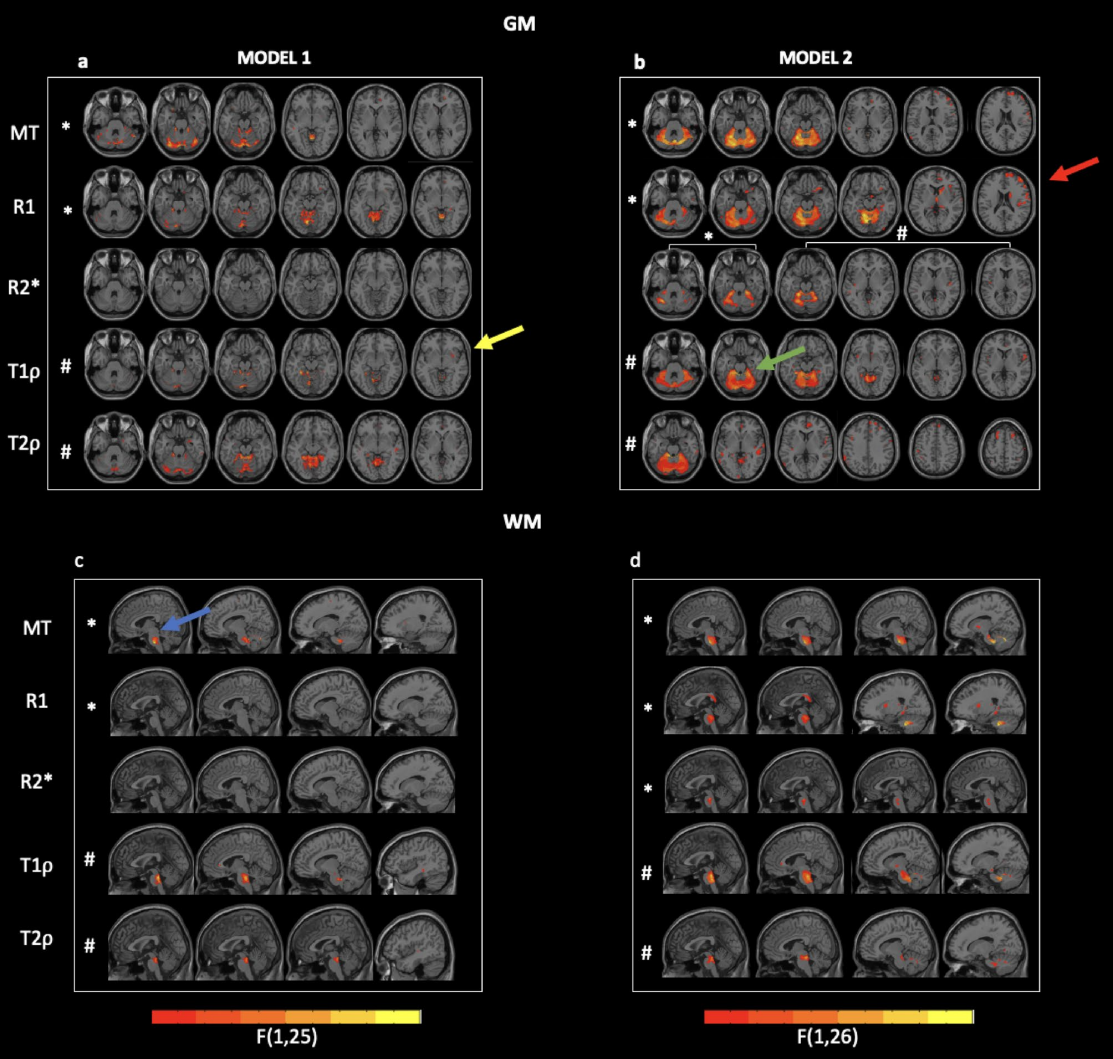
F-maps of statistically significant group effects in all parameters as obtained from the model 1 (panel a and c) and model 2 (panel b and d) in the GM (panels a and b) and in the WM (panels c and d). Clusters with an extension higher than 30 voxels are overlaid to axial or sagittal cuts from the T1-w image. Cluster are reported at a p-value at the peak below 5% (FDR-corrected for multiple comparisons). Bars are only representative of the trend from lower (orange) to higher (yellow) F values. Yellow arrow highlights the Putamen cluster, red arrow the frontal regions, green the cerebellum and blue the pons. * Symbol represents the comparison HC > MSA, # symbol represents the comparison HC < MSA.

When the GM tissue volume was included in the model (1), clusters of significant group effects were detected for most parameters except R2* (Fig. 3a and Supplementary Table 1). A closer inspection of the clusters exhibiting such group effects revealed smaller MT in the brain of MSA patients in the posterior cerebellum, smaller R1 in the anterior and posterior cerebellum, middle temporal gyrus, superior frontal cortex, and limbic area, longer T1ρ in the anterior cerebellum, the left fusiform area, and the right putamen of MSA patients and longer T2ρ in the posterior cerebellum, the left and right anterior cerebellum, temporal and frontal areas.

When the GM tissue volume was not included in the model (2), clusters of significant group differences were detected for all parameters (Fig. 3b and Supplementary Table 2). In particular, MSA patients exhibited smaller MT in the anterior cerebellum, frontal, pre- and postcentral gyri, along with smaller R1 in numerous regions encompassing the anterior cerebellum, all cortical lobes, and deep brain structures such as thalamus, hippocampus and caudate. MSA patients also had larger R2* in the insula and smaller R2* in cerebellum. As for the RFR, significant increases in T1ρ were found in the anterior cerebellum, cingulate, and frontal cortex of MSA patients, whereas significant increases in T2ρ were found in the cingulate, frontal, parietal, and occipital cortices.

### Voxel-wise analysis in white matter

When the WM tissue volume was included in the model (1), we detected statistically significant effects only for MT, T1ρ, and T2ρ in the pons and, for the MT saturation parameter, in the frontal cortex (Fig. 3c and Supplementary Table 3).

When tissue volume was not included in the model (2), clusters of significant group effects were detected for all parameters (Fig. 3d and Supplementary Table 4). In particular, smaller MT was detected in the pons, sub-lobar regions, and right frontal region of MSA patients, along with smaller R1 in the pons, the parahippocampal region, bilateral sub-lobar region, corpus callosum and right frontal region. In addition, longer T1ρ were detected in the pons and in the right temporal lobe, while longer T2ρ were found in the pons, cerebellum, and temporal lobe.

### ROI analyses

**Fig. 4:**
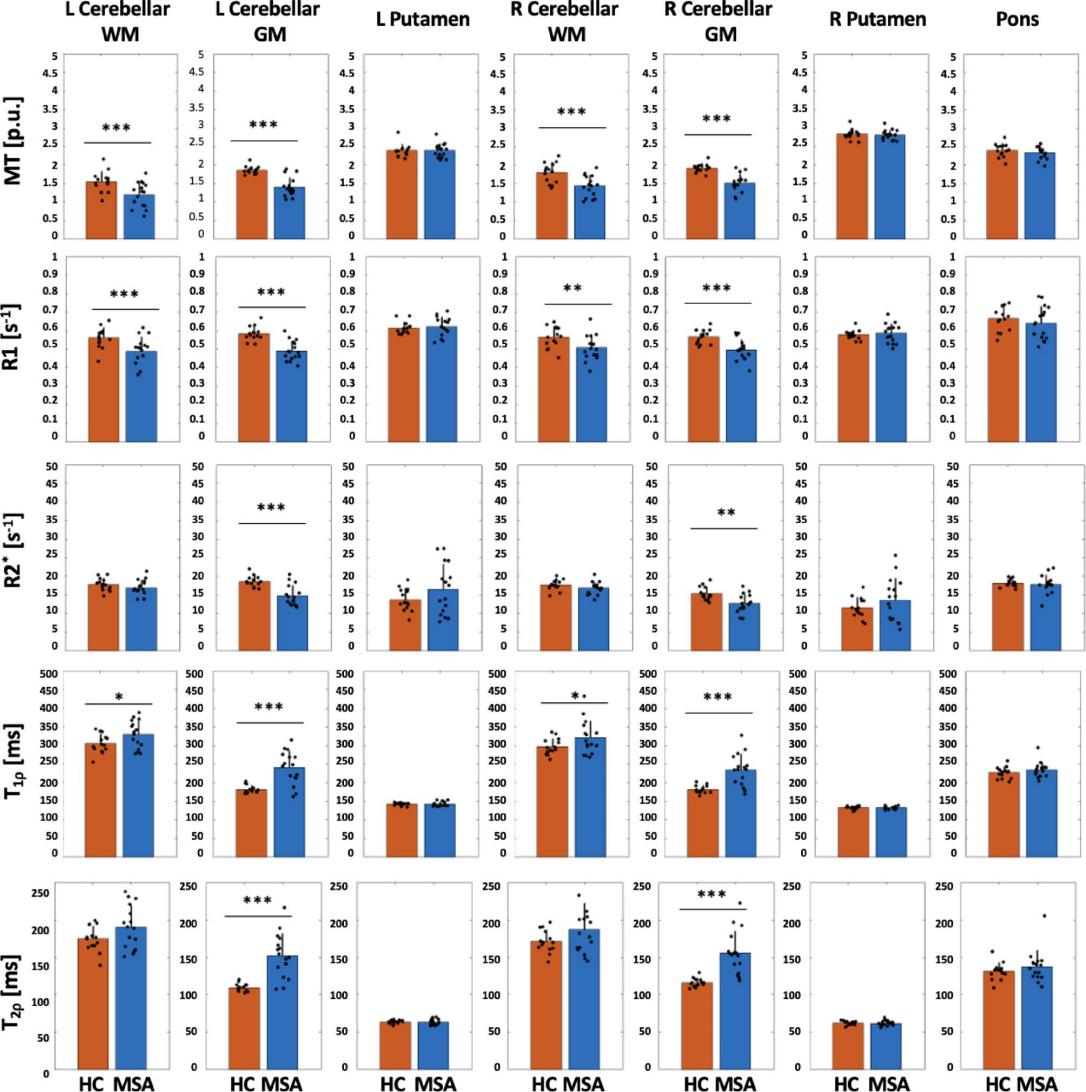
Bar plots summarizing the ROI data distributions in each group after regressing out age, gender and tissue volume (model 1). Bars display group mean ± S.D., and points represent the individual data. Significant group effects obtained from the LME analysis are indicated with asterisks: * *p* < 0.05, ** *p* < 0.01 and *** *p* < 0.001.

**Fig. 5:**
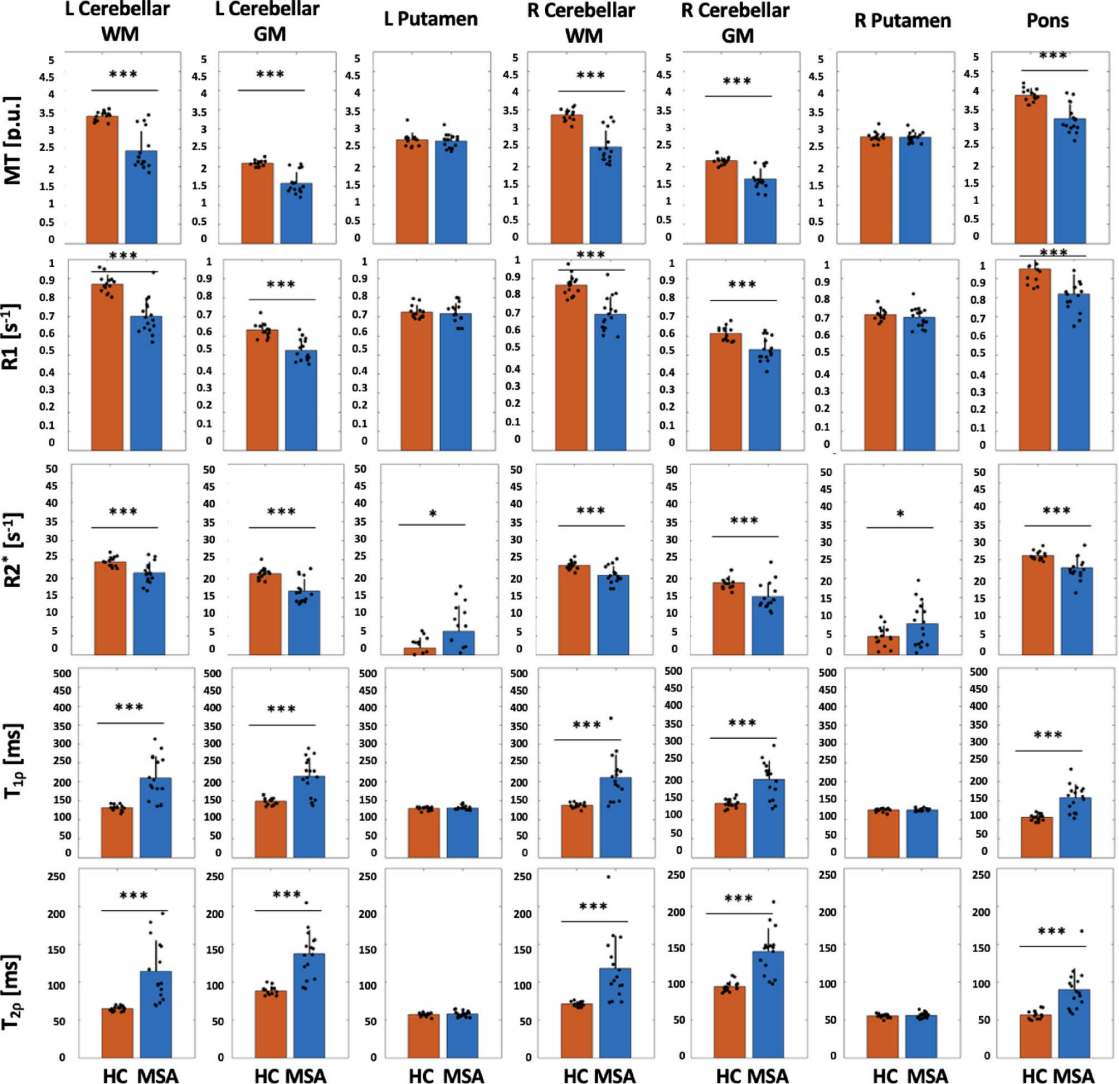
Bar plots summarizing the ROI data distributions in each group after regressing out age and gender (model 2). Bars display group mean ± S.D., and points represent the individual data. Significant group effects obtained from the LME analysis are indicated with asterisks: * *p* < 0.05, ** *p* < 0.01 and *** *p* < 0.001.

The results of the ROI analysis are summarized in Table 2. The data distributions and the statistically significant group effects resulting from the models 1 and 2 applied to the mean values within the ROIs are reported in Figs. 4 and 5 (where we respectively plotted the data after regressing out age, gender and tissue volume and the data after regressing out age and gender). The findings are generally consistent with observations resulting from the voxel-wise analyses (Figs. 3), albeit some differences also exist due to the inherently different sensitivities of the two approaches. In particular, the ROI analyses confirmed significant group effects for most metrics in the cerebellum and in the pons that were not detected after accounting for tissue volume in the model. On the other hand, the ROI analysis did not detect group effects for any metric in the putamen, in contrast to what resulted from the voxel-wise analysis that instead revealed a significant group effect (due to a longer T1ρ) in the right putamen of MSA. 

**Table 2:** F- and p-values obtained from the ROI analysis computed with and without considering the effect of tissue volume (model 1 and model 2, respectively).

Region	Parameter	*Model 1*		*Model 2*	
		F(1,25)	p	F(1,26)	p
Left cerebellar WM	MT	**23.08**	**0.0001**	**53.83**	2.01*10 ^**−7**^
	R1	**19.37**	**0.0004**	**48.05**	1.63*10 ^**−6**^
	R2*	2.87	0.12	**15.38**	**0.001**
	T1ρ	**8.58**	**0.02**	**30.97**	1.78*10 ^**−5**^
	T2ρ	5.69	0.06	**24.31**	7.06*10 ^**−5**^
Left cerebellar GM	MT	**55.90**	5.52*10 ^**−7**^	**54.44**	2.01*10 **−7**
	R1	**42.22**	7.1*10 − ^**6**^	**43.43**	1.91*10 ^**−6**^
	R2*	**27.56**	**0.0001**	**28.13**	**0.0001**
	T1ρ	**29.27**	9.07*10 ^**−5**^	**31.09**	1.78*10 ^**−5**^
	T2ρ	**38.37**	1.24*10 ^**−5**^	**40.73**	6.12*10 ^**−6**^
Right cerebellar WM	MT	**29.50**	2.9*10 ^**−5**^	**61.89**	1.69*10 ^**−7**^
	R1	**12.77**	**0.003**	**36.60**	5.06*10 ^**−6**^
	R2*	3.93	0.08	**18.32**	**0.0008**
	T1ρ	**6.37**	**0.03**	**24.77**	5.0*10 ^**−5**^
	T2ρ	4.35	0.08	**18.72**	**0.0003**
Right cerebellar GM	MT	**46.61**	1.30*10 ^**−6**^	**46.57**	5.32*10 ^**−7**^
	R1	**20.23**	**0.0004**	**22.95**	**0.0001**
	R2*	**14.78**	**0.003**	**14.82**	**0.001**
	T1ρ	**26.20**	9.60*10 ^**−5**^	**27.54**	3.06*10 ^**−5**^
	T2ρ	**35.01**	1.24*10 ^**−5**^	**37.61**	6.12*10 ^**−6**^
Pons	MT	3.18	0.12	**38.86**	1.89*10 ^**−6**^
	R1	1.79	0.27	**18.73**	**0.0003**
	R2*	0.51	0.48	**16.77**	**0.0008**
	T1ρ	3.36	0.11	**36.23**	1.64*10 ^**−5**^
	T2ρ	2.52	0.17	**24.72**	7.06*10 ^**−5**^
Left Putamen	MT	0.00	0.96	0.47	0.58
	R1	0.46	0.50	0.23	0.64
	R2*	4.51	0.08	**5.98**	**0.025**
	T1ρ	0.06	0.80	0.57	0.48
	T2ρ	0.00	0.95	0.51	0.48
Right Putamen	MT	0.59	0.52	0.08	0.78
	R1	0.81	0.44	1.24	0.32
	R2*	4.71	0.08	**4.33**	**0.047**
	T1ρ	0.24	0.73	0.52	0.48
	T2ρ	0.12	0.85	0.87	0.42

Differences are considered significant (and indicated in bold) after correction for multiple comparisons using FDR

### Correlation analyses

When the ROI data were corrected for age and gender, significant correlations were observed only between the T1ρ in the right cerebellar GM and disease duration (ρ = 0.55, *p* = 0.03). When the data were corrected for age, gender, and volume, significant correlations were found between MT values in the right cerebellar GM and disease duration (ρ=−0.51, *p* = 0.04), between R2* in the left cerebellar WM and UMSARS II (ρ=−0.56, *p* = 0.02), between T1ρ values in the right cerebellar GM and disease duration (ρ = 0.66, *p* = 0.005) and between T2ρ values both in both the right and left cerebellar GM and disease duration (ρ = 0.71, *p* = 0.002; ρ = 0.51, *p* = 0.04, respectively).

## Discussion

In this work, we measured quantitative and semi-quantitative MRI parameters, namely MT, R1, R2*, adiabatic T1ρ, and T2ρ, and performed a cross-sectional study to characterize differences in the brain of MSA patients as compared to HC subjects, with an additional focus on the effects of brain atrophy, known to be present and mostly diffuse in MSA^24^.

The first result of this study was that significant brain atrophy was observed in the MSA patients in the cerebellum white and grey matter and in the frontal white matter, albeit only when correction for multiple comparisons was performed at a cluster level and at a lower statistical threshold. These results are likely due to the small sample size used in this study and to the mixed nature of the MSA group.

Subsequently, we investigated the between-group differences in several quantitative and semi-quantitative MRI parameters and analyzed the effect of tissue volume on those group effects. Generally, at both voxel and regional levels, we found that the differences between groups were more spread in the brain when not including tissue volume/ROI volume as a confounding effect, but even when we accounted for this effect, alterations remained statistically significant in brain regions specifically targeted by MSA pathology (i.e., cerebellum and putamen). Furthermore, when accounting for tissue volume, some additional differences emerge, like in the case of a cluster in the right putamen revealed in T1ρ analysis. This finding, which highlights an increase of T1ρ in MSA patients vs. HC, might be related to the accumulation of alpha-synuclein aggregates in this region^25^. Indeed, previous findings in Alzheimer’s disease have reported that macromolecular protein aggregation prolongs T1ρ^26^, supporting the idea that putaminal T1ρ may be an adjunctive quantitative biomarker of MSA pathology. However, this effect was not detected in the ROI analysis, suggesting this alteration was a localized effect involving only a portion of the anatomical structure.

Similarly to what observed in the voxel-wise analysis, also at the regional level, the inclusion of the ROI volume term in the LME models determined a reduction of the number of significant differences observed on the MRI parameters: significant effects were scattered within the putamen and the bilateral cerebellar WM for R2*, in the bilateral cerebellar WM for T2ρ, and in the pons for all parameters. In regions where microstructural abnormalities occur concordantly with macrostructural alterations resulting in atrophy, it is not surprising that accounting for atrophy may abolish group differences in microstructural parameters. Indeed, subtle macrostructural abnormalities in large ROIs might be lost just because of partial volume effects, without implying that there are no group differences. Interestingly, when tissue volume is accounted for, cerebellar MT, R2* and T2ρ were found to correlate with disease duration and these correlations may indicate that these quantitative parameters may serve as markers of disease progression and its related brain changes.

The reduction observed for the MT and R1 parameters in the cerebellar regions has been previously linked to alterations in the myelin structure^27^ in both the WM and GM^28^. Furthermore, increased T2ρ in the cerebellar GM is opposite to what was observed in the cerebellum of Parkinson’s disease patients, where a shorter T2ρ was found in patients as compared to HC^11^. Nonetheless, this is consistent with previous findings on the cerebellum of patients with primary progressive multiple sclerosis^14^. The similarity between MSA and primary progressive multiple sclerosis could be explained by the fact that MSA can be considered a primary oligodendrogliopathy^29^, thereby T2ρ sequences would be able to capture such macromolecular alterations during the course of neurodegeneration, which results in prolongation of T2ρ time constants due to myelin disruption followed by axonal dysfunction and ultimately in gliosis. When not considering tissue volume as confounding factor in the statistical model, group differences emerged as significant in the pons (for all parameters) and in the bilateral putamen for R2*, which was found to be increased in the MSA population, in line with previous studies reporting iron accumulation^30,31^.

To the best of our knowledge, this is the first study applying RFR metrics for the study of MSA pathology. Previous findings have been reported with the more conventional relaxation-weighted MRI parameters (i.e., T1w or T2w sequences) or with a more classical relaxometry approach. For example, in the paper by Tzarouchi et al.^32^, MSA patients showed GM atrophy in the putamen, caudate, thalami, anterior cerebellar lobes, and the cerebral cortex, white matter atrophy in pons, midbrain, and peduncles, and prolonged T2 bilaterally in various cortical regions and in the posterior cerebellar lobes, which ceased to be significant after controlling for gray and white matter volume. Specht et al.^33^ revealed a reduction of R2 in the cerebellum and brainstem but also an increase in the putamen with all the affected regions largely corresponding to those showing GM or WM reduction by the VBM. Our study suggests that the combination of voxel-based relaxation and atrophy effects in the same analysis may provide either complementary or ancillary information about the brain morphology, depending on how the two effects interact with each other, albeit occurring at different physical (spatial or temporal) scales. In the work of Naka et al.^34^, the MT rate was altered at the base of the pons, the middle cerebellar peduncle, in the putamen, and in the white matter at the level of the precentral gyrus, resulting in significantly lower rate in the MSA patients compared to the HC subjects. However, some discrepancies among reported results exist for this disease. Indeed, Focke et al.^35^, comparing the MSA-P subtype with a control group, found significant increases of R2* in the putamen bilaterally and the right Globus Pallidum but no other significant differences were detected in the MT, MTR, R1, or R2 maps. Our results are overall in line with previous literature, even though a strict comparison with previous works should be done with caution since only a few studies accounted for the possible effect of atrophy on the differences emerging for the qMRI parameters.

Even if the present results pertain to a single-center study, the quantitative nature of the investigated qMRI parameters will enable direct future comparisons with data obtained in other centers. Collection of data from multiple centers is expected to be straightforward for adiabatic T1ρ and T2ρ, which are inherently resilient to radiofrequency (RF) transmit (B1 +) inhomogeneities. Yet, B1 + inhomogeneities can impact the MT and R1 mapping, and they thus need to be taken into account in multi-center studies. The opportunity of performing qMRI in a multi-center fashion will enable a more physically meaningful, and possibly clinically effective, interpretation of the observed changes^36^. In contrast to simply assessing tissue atrophy via structural image registration and segmentation of T1- and T2-weighted 3D images, the qMRI approach proposed here should allow the investigation of biologically distinct microstructural processes, which may in principle precede changes in tissue volume and morphology manifesting as atrophy^37^. For example, macromolecular and biochemical changes in tissue can be quantified using RFR metrics.

However, the MPM protocol was chosen for its benefit of providing high level of consistency across sites^38^ and its ability to produce whole-brain maps of R1, MT, and R2* at high spatial resolution (1 mm isotropic) within a clinically feasible scan time of approximately 24 min. This quantitative imaging approach allows for reliable assessment of microstructural tissue properties such as myelination, iron content, and macromolecular composition^39^. The MPM protocol has been successfully applied not only in single-site imaging studies but also in multi-site settings, demonstrating high levels of consistency and reproducibility across different scanners and sites^38^. As described in^39^, the sequence parameters were carefully selected to achieve an optimal balance between signal quality, tissue contrast, and acquisition efficiency for mapping all three parameters. A short acquisition time was made possible by combining GRAPPA parallel imaging (acceleration factor of 2) with Partial Fourier acquisition.

In addition, for reliable R2* estimation, the echo train length was limited to about 20 ms. This represented a practical compromise, enhancing R2* contrast while minimizing signal loss due to susceptibility effects, especially in areas with steep magnetic field gradients^40^. This setup also allowed for echo averaging, which improved the overall signal-to-noise ratio (SNR). Additionally, a high readout bandwidth was used to reduce off-resonance effects and chemical shift artifacts^18^.

It is well known that R2* values are affected by magnetic field inhomogeneities (see for example^41^). However, as part of the hMRI tool^22^, used for processing the images acquired from the MPM protocol for the R2* mapping, a robust log-linear (or ESTATICS) fit of signal versus echo time across the six echoes of the gradient-echo readout was performed. Because this fit models the decay due to both true T₂ relaxation and reversible dephasing from local B₀ gradients, it inherently reduces the static field inhomogeneity into the R₂* estimate rather than requiring a separate B₀ correction step for the R₂* map^42^. In addition, high spatial resolution (1 mm isotropic) of the acquired maps further minimizes within-voxel susceptibility artefacts (smaller phase dispersion), improving the robustness of the mono-exponential fit.

Our findings should also be interpreted in light of the known vascular contributions to the R2* signal^43^. Indeed, as known, R2* is sensitive not only to microstructural properties of brain tissue but also to changes in the oxygen extraction fraction (OEF), a key determinant of the BOLD signal. This implies that group differences in R2*, such as those observed between healthy controls and MSA patients, may not solely reflect alterations in tissue architecture or iron content, but could also be influenced by underlying differences in cerebral metabolism. Given that neurodegenerative diseases like MSA are associated with impaired mitochondrial function and altered metabolic demands^44,45^, it is plausible that the observed R2* changes partially stem from a disrupted balance between oxygen delivery and consumption. Thus, metabolic contributions to R2* contrast should be carefully considered, particularly when interpreting R2* as a purely structural or iron-sensitive biomarker. Future studies combining R2* mapping with complementary metabolic imaging techniques (e.g., calibrated fMRI or PET) may help disentangle these effects and provide a more comprehensive understanding of the pathophysiological mechanisms at play.

This study has some limitations that need to be acknowledged. MSA is a rare disease, which limits the access to large patient populations for a single-center study, yet bigger sample sizes with a more balanced distribution of gender and MSA subtypes are needed to validate the statistical differences observed in this work and to gain further insights regarding the differences between MSA subtypes on the observed tissue abnormalities. In addition, the cross-sectional design of the study is suboptimal, because the lack of a longitudinal comparison limits the assessment of a possible predictive nature of these metrics and the resulting added benefit for prognosis. Future investigations are needed especially towards understanding whether and when the evolution of microstructural changes may lead to macrostructural changes. Finally, in order to maintain an acceptable scan time, especially considering the clinical population under investigation, the brain coverage of T1ρ and T2ρ acquisitions was smaller than the one achieved in the MPM protocol. Lastly, although adiabatic T1ρ and T2ρ techniques are resilient to B1 alterations since the relaxation time constants having minor B1 dependence, yet an addition to the acquisition protocol of B1 mapping will be beneficial for more precise determination of qMRI metrics.

## Conclusions

In summary, our cross-sectional MRI data in MSA indicate that adiabatic rotating‐frame relaxometry metrics (T1ρ and T2ρ) reveal microstructural tissue changes that go beyond what conventional R1, R2*, or MT may detect. Notably, T1ρ and T2ρ group differences remained significantly different even after correcting for regional atrophy, suggesting that these measures indicate intrinsic tissue pathology (e.g., changes in myelin density and/or integrity, or cellular integrity) rather than merely reflecting volume loss. This is consistent with the known biophysics of rotating‐frame MRI metrics, which probe intermediate‐to‐slow motional regimes with higher sensitivity than conventional T1 and T2^46^. In particular, T1ρ is highly sensitive to low‐frequency molecular motions and macromolecular interactions in the in the ms time scale, while T2ρ is exceptionally sensitive to proton exchange and/or diffusion in iron‐rich environments with the sensitivity shifted to ms-ms time scale. In practical terms, elevated T1ρ can reflect pathological processes such as demyelination/myelin disruption, neuronal loss, and gliosis, and T2ρ can reflect alterations in iron content and metabolism. Thus, T1ρ and T2ρ provide complementary contrasts to traditional MRI – capturing subtle biochemical and microstructural changes that may precede gross atrophy. These findings highlight the promise of rotating‐frame metrics as novel biomarkers for MSA, with potential utility in early disease detection and in monitoring progression independent of overt tissue loss.

## Electronic supplementary material

Below is the link to the electronic supplementary material.

Supplementary Material 1

## Data Availability

Raw data used in the study are available from the corresponding author upon reasonable request.
