# Supplementary material for: Microstructural abnormalities in multiple system atrophy as revealed by conventional and rotating frame relaxation MRI parameters
Source: Sci Rep. 2025 Jul 24;15:26954. doi: 10.1038/s41598-025-10812-6 (PMC12289937; doi:10.1038/s41598-025-10812-6)
Supplement: Supplementary file 1 — Supplementary Material 1 [file 41598_2025_10812_MOESM1_ESM.docx]

**MRI data acquisition**

MRI data were acquired on a 3 T MRI scanner (MAGNETOM Skyra, Siemens, Erlangen Germany) equipped with a 20-channel radio-frequency (RF) receive head coil and body RF transmit coil.

The imaging protocol consisted of: a 3D anatomical T1-weighted Magnetization Prepared RApid Gradient Echo (MPRAGE) sequence with TR/TE = 2400/2.25 ms, resolution = 1 × 1 × 1 mm^3^, matrix size = 256 × 256, anterior-posterior phase encoding direction, generalized auto-calibrating partially parallel acquisitions (GRAPPA) factor of 2 in phase-encoding direction, bandwidth 200 Hz/Px, non-selective excitation; three different multi-echo 3D FLASH sequences (MPM protocol) with predominant T1-, proton density (PD)-, and MT-weighting by appropriate choice of the TR and the flip angle α (TR/α = 18.7 ms/20° for the T1w scan and 23.7 ms/6° for the PDw and the MTw scans) [1]. The MT-weighting was obtained by applying an off-resonance RF pulse (4 ms duration, 220° nominal flip angle, 2 kHz frequency offset from water resonance) before the RF excitation. Multiple gradient echoes were acquired with alternating readout polarity at six equidistant TEs between 2.4 and 14.7 ms for the MTw, with two additional TEs at 17.2 and 19.6 ms for the PDw and T1w acquisition. Multi-echo FLASH data allow for R2* mapping and, via averaging, allows increasing the SNR of R1 and MT maps in comparison with single-echo FLASH data. All the MPM sequences were acquired at 1 mm isotropic resolution, matrix size = 256 × 240 × 176, GRAPPA factor of 2 in phase-encoding direction, and no partial Fourier reconstruction. Adiabatic T1ρ, T2ρ measurements were collected from 35 AC-PC aligned slices using a segmented GRE readout with 4 segments, voxel size = 1.6 × 1.6 × 3.6 mm^3^, GRAPPA= 3, TE = 3.18 ms and TR = 2 s. For adiabatic T1ρ and T2ρ measurements, hyperbolic secant (HS) pulses with stretching factor = 1 were used with BW = 1.6 kHz, pulse duration Tp = 6 ms, and peak power ωmax /(2π) = 800 Hz. A train with incremental number of such HS pulses = 0, 4, 8, 12, 16 phase-cycled according to the MLEV4 scheme [2–4] was placed right before the readout. In T2ρ acquisitions, the train of HS pulses was further embedded within two adiabatic half passage (AHP) pulses for placing magnetization on the transverse plane and back to the longitudinal axis before the readout.

**Preprocessing of MPM maps**

To increase the signal-to-noise ratio, arithmetic mean images from the first six shortest TE acquisitions were calculated for each FLASH series to produce T1w, PDw, and MTw volumes. These three mean images were used to obtain the MT and the apparent longitudinal relaxation rate (R1app) maps. Here, the MT metric is a semi-quantitative parameter which reflects a measure of MT saturation, defined as the percentage saturation imposed by one MT pulse during TR. This parameter is expressed in percent unit and is calculated according to the procedure explained in [5]. Quantitative R1 maps were estimated from R1app maps by correcting for RF transmit bias using the UNICORT method [6]. R2* maps were obtained with the ESTATICS method [7] which is based on a log-linear regression model applied to all echo images from the MPM series (PD-, T1-, and MT-weighted). This procedure allows all data to be pooled while still accounting for the varying contrast and has been shown to provide increased robustness to motion artifacts [7].

**Creation of masks for the voxel-wise analysis**

To assess the differences in terms of tissue volume between the two populations and to normalize the obtained relaxometry maps in the MNI space to perform the voxel-wise group analyses, T1w images were first segmented into the three main tissues, grey matter (GM), white matter (WM), and cerebrospinal fluid (CSF), and then normalized to MNI standard space using the DARTEL approach [8]. To assess the differences in terms of atrophy between healthy controls and MSA patients, the resulting gray matter and white matter probabilistic maps were modulated by the Jacobian determinants of the deformations to account for local compression and expansion due to linear and non-linear transformation [9] and then smoothed with a Gaussian kernel of 6 mm FWHM. For the latter purpose, all smoothed maps were resampled to a voxel size of 2 × 2 × 2 mm^3^. From this segmentation, the total intracranial volume was also estimated for each subject using the SPM12 utility function “tissue volumes”. The deformation fields obtained from the above-described DARTEL procedure were used to normalize to the MNI space all the obtained relaxometry maps, after co-registration of these maps with the anatomical T1w images. The normalized maps were resampled to a voxel size of 2 × 2 × 2 mm^3^.

To differentially smooth the maps in the GM and WM tissues, group masks of these two primary tissues were generated from the GM and WM maps of the entire study group: for each subject, each voxel was classified as GM, and its value was set to 1, if the probability value of the GM map in that voxel was higher than the probability value of the WM map in that voxel and if that value was higher than 0.2; vice versa, the voxel was classified as WM, and its value was set to 1, if the probability value of the WM map in that voxel was higher than the probability value of the GM map in that voxel and if that value was higher than 0.2. All the binarized GM and WM masks obtained above (a set of two masks for each subject) were then summed and only the voxels showing the maximum value corresponding to the size of the entire group were respectively considered in the final WM and GM binarized group masks [10]. To consider the common area of acquisitions across all the MR parameters and subjects, we created an additional binary mask (referred to as coverage mask) in the MNI space covering the area of acquisition of the T1ρ and T2ρ maps, whose FOV was smaller than the one set for the other acquisitions. For the statistical comparisons, the masks of GM and WM were multiplied with the coverage mask to restrict the analyses to the common voxels only. Using these masks and the anatomically informed smoothing function implemented in the hMRI tool, all maps were smoothed with a Gaussian kernel with isotropic FWHM of 6 mm.

**Creation of masks for the ROIs analysis**

To extract the masks of the a priori ROIs, the T1w images were processed using the “recon-all” algorithm from the Freesurfer v 6.0 library (<https://surfer.nmr.mgh.harvard.edu>). Regions in the cerebellar left and right WM and GM and in the left and right putamen were extracted from the resulting “aseg+aparc” segmentation. To obtain an ROI in the pons, the brainstem-structures algorithm (additional module implemented in Freesurfer 6.0) was also used. Data from multi-echo flash and adiabatic T1ρ/T2ρ acquisitions were coregistered to the high-contrast T1w images using the FLIRT command of FSL (https://fsl.fmrib.ox.ac.uk/fsl/). The transformation matrices were then inverted and applied to the ROI using the nearest neighbor interpolation in such a way to have the mean values being extracted in the native space of the considered maps. As in the voxel-based analysis, in the ROI-based analysis, with the goal of considering differences in the acquisition FOV across subjects that may affect the statistical results, we first multiplied the atlas for the coverage masks that were brought back into the native space.

**Analysis of the GM considering tissue volume as a fixed effect in the LME model (model 1)**

| **MT** | | | | | | |
| --- | --- | --- | --- | --- | --- | --- |
| **Region** | **# of voxels** | **x** | **y** | **z** | **F_peak_**  **P_Peak_** | **Comparison direction** |
| L Posterior cerebellum | 2565 | -2 | -64 | -12 | 59.35  4.64*10-8 | HC>MSA |
| **R1** | | | | | | |
| L anterior cerebellum | 1351 | -8 | -68 | -14 | 48.27  2.78*10-7 | HC>MSA |
| L posterior cerebellum | 62 | -38 | -78 | -26 | 20.06  0.003 | HC>MSA |
| L middle temporal | 56 | -52 | -36 | 0 | 20.90  0.0001 | HC>MSA |
| R Limbic area | 31 | 12 | 46 | 4 | 28.32  1.62*10-5 | HC>MSA |
| R middle temporal gyrus | 41 | 44 | 50 | 20 | 29.31  1.28*10-5 | HC>MSA |
| R superior frontal | 30 | 16 | 34 | 50 | 27.53  1.97*10-5 | HC>MSA |
| **R2*** | | | | | | |
| No clusters | | | | | | |
| **T1ρ** | | | | | | |
| L anterior cerebellum | 383 | -8 | -58 | -8 | 40.1  1.26*10-6 | HC<MSA |
| L Fusiform | 95 | -30 | -40 | -16 | 42.8  7.41*10-7 | HC<MSA |
| R Putamen | 30 | 32 | 8 | -2 | 26.5  2.54*10-5 | HC<MSA |
| **T2ρ** | | | | | | |
| L anterior cerebellum | 40 | -36 | -46 | -36 | 22.44  7.37*10-5 | HC<MSA |
| R anterior cerebellum | 2320 | 6 | -40 | -18 | 90.61  8.59*10-10 | HC<MSA |
| R limbic lobe | 38 | 30 | 2 | -24 | 19.32  0.0003 | HC<MSA |
| R posterior cerebellum | 60 | 26 | -76 | -24 | 18.97  0.0002 | HC<MSA |
| R superior temporal | 32 | 52 | -20 | -6 | 18.1  0.0003 | HC<MSA |
| R supero-medial frontal | 58 | 8 | 58 | 28 | 19.49  0.0002 | HC<MSA |
| R superior frontal | 39 | 18 | 58 | 24 | 18.05  0.0003 | HC<MSA |
| L supramarginal gyrus | 34 | -62 | -54 | 32 | 17.17  0.0003 | HC<MSA |

**Supplementary Table 1**: Regions of statistically significant differences in GM for each metric, obtained from the LME analysis that considers the effect of atrophy in the model. The statistical threshold is p<0.05 FDR corrected. The minimum cluster size is equal to 30 voxels. The number of voxels in the cluster are reported along with the MNI coordinate, the F- and p-values of the statistical peak of the difference.

**Analysis of the GM without considering tissue volume as a fixed effect in the LME model (model 2)**

| **MT** | | | | | | |
| --- | --- | --- | --- | --- | --- | --- |
| **Region** | **# of voxels** | **x** | **y** | **z** | **F_peak_**  **P_Peak_** | **Comparison direction** |
| L anterior cerebellum | 6846 | -24 | -56 | -22 | 73.65  4.62*19-9 | HC>MSA |
| R anterior cerebellum | 61 | 8 | -56 | -34 | 57.50  4.75*10-8 | HC>MSA |
| R superior frontal gyrus | 156 | 20 | 64 | 22 | 17.72  0.0003 | HC>MSA |
| R middle frontal | 58 | 42 | 46 | 16 | 16.55  0.0004 | HC>MSA |
| R postcentral gyrus | 44 | 66 | -6 | 24 | 23.90  4.5*10-5 | HC>MSA |
| R precentral | 193 | 50 | -8 | 44 | 26.64  2.20*10-5 | HC>MSA |
| R middle frontal | 65 | 38 | 32 | 44 | 20.65  0.0001 | HC>MSA |
| L precuneus | 32 | -36 | -76 | 38 | 19.16  0.0002 | HC>MSA |
| L middle frontal gyrus | 64 | -40 | 4 | 56 | 19.31  0.0002 | HC>MSA |
| L superior frontal gyrus | 37 | -12 | 36 | 54 | 16.42  0.0004 | HC>MSA |
| R superior frontal | 59 | 18 | 36 | 52 | 18.39  0.0002 | HC>MSA |
| **R1** | | | | | | |
| L anterior cerebellum | 6283 | -10 | -68 | -14 | 47.33  2.65*10-7 | HC>MSA |
| R anterior cerebellum | 68 | -10 | -56 | -36 | 21.89  7.86*10-5 | HC>MSA |
| R inferior frontal gyrus | 187 | 30 | 20 | -10 | 22.51  6.59*10-5 | HC>MSA |
| R inferior occipital gyrus | 46 | 36 | -90 | -18 | 14.52  0.0008 | HC<MSA |
| R Caudate | 483 | 12 | 6 | 6 | 21.48  8.83*10-5 | HC>MSA |
| R hippocampus | 296 | 24 | -32 | -4 | 21.07  9.92*10-5 | HC>MSA |
| R thalamus | 164 | 2 | -10 | 2 | 18.08  0.0002 | HC>MSA |
| L hippocampus | 69 | -18 | -34 | -2 | 13.82  0.001 | HC>MSA |
| R supero-medial frontal | 74 | 12 | 46 | 0 | 17.17  0.0003 | HC>MSA |
| L middle temporal | 106 | -54 | -36 | 0 | 18.66  0.0002 | HC>MSA |
| R inferior frontal | 79 | 40 | 22 | 2 | 12.70  0.001 | HC>MSA |
| R middle frontal | 877 | 42 | 46 | 18 | 20.00  0.0001 | HC>MSA |
| R olandic operculum | 139 | 52 | -18 | 20 | 14.83  0.0007 | HC>MSA |
| R precentral gyrus | 597 | 44 | -10 | 40 | 27.12  1.94*10-5 | HC>MSA |
| R inferior parietal | 90 | 60 | -30 | 30 | 34.01  3.80*10-6 | HC>MSA |
| L inferior parietal | 179 | -66 | -36 | 32 | 19.56  0.0001 | HC>MSA |
| R middle cingulate | 52 | 0 | -30 | 30 | 10.88  0.003 | HC>MSA |
| R middle cingulate | 41 | 6 | 34 | 30 | 10.83  0.003 | HC>MSA |
| L superior frontal | 37 | -26 | 36 | 34 | 15.63  0.004 | HC>MSA |
| L postcentral | 42 | -62 | -14 | 36 | 10.02  0.004 | HC>MSA |
| L precuneus | 58 | -38 | -80 | 40 | 17.05  0.0003 | HC>MSA |
| R middle frontal | 84 | 40 | 28 | 50 | 26.12  2.51*10-5 | HC>MSA |
| L postcentral | 139 | -50 | -26 | 48 | 18.56  0.0002 | HC>MSA |
| L middle frontal | 141 | -40 | 4 | 58 | 15.32  0.0006 | HC>MSA |
| R superior frontal | 115 | 18 | 38 | 54 | 22.23  7.13*10-5 | HC>MSA |
| L superior frontal | 40 | -12 | 36 | 54 | 13.67  0.001 | HC>MSA |
| R middle frontal | 35 | 40 | 8 | 58 | 11.27  0.002 | HC>MSA |
| R superior frontal | 63 | 24 | 8 | 56 | 18.66  0.0002 | HC>MSA |
| L superior frontal | 48 | -20 | 14 | 66 | 13.79  0.001 | HC>MSA |
| L paracentral lobule | 57 | -4 | -24 | 66 | 14.37  0.0008 | HC>MSA |
| R superior frontal | 103 | 22 | 0 | 68 | 17.87  0.0002 | HC>MSA |
| **R2*** | | | | | | |
| L anterior cerebellum | 2974 | -18 | -48 | -16 | 36.70  2.12*10-6 | HC>MSA |
| L insula | 48 | -44 | -12 | 10 | 16.37  0.0004 | HC<MSA |
| **T1ρ** | | | | | | |
| L anterior cerebellum | 7025 | -4 | -44 | -22 | 51.68  1.24*10-7 | HC<MSA |
| R anterior cerebellum | 65 | 0 | -54 | -34 | 21.98  7.65*10-5 | HC<MSA |
| L insula | 44 | -44 | 4 | 0 | 12.05  0.002 | HC<MSA |
| R inferior frontal operculum | 216 | 56 | 10 | 10 | 18.33  0.0002 | HC<MSA |
| L inferior frontal gyrus | 55 | -50 | 18 | 20 | 15.18  0.0006 | HC<MSA |
| R supero-medial frontal | 57 | 10 | 60 | 14 | 16.01  0.0005 | HC<MSA |
| R superior frontal gyrus | 53 | 22 | 56 | 26 | 13.45  0.001 | HC<MSA |
| R anterior cingulate | 39 | 8 | 42 | 24 | 14.50  0.0008 | HC<MSA |
| R supramarginal gyrus | 37 | 56 | -56 | 34 | 13.25  0.002 | HC<MSA |
| **T2ρ** | | | | | | |
| L anterior cerebellum | 7380 | 6 | -40 | -18 | 88.55  7.40*10-10 | HC<MSA |
| L anterior cerebellum | 68 | -2 | -60 | -36 | 26.91  2.05*10-5 | HC<MSA |
| R limbic lobe | 64 | 24 | 2 | -30 | 16.76  0.0004 | HC<MSA |
| R Middle temporal gyrus | 72 | 64 | -26 | -18 | 12.59  0.001 | HC<MSA |
| R occipital lobe | 30 | 2 | -90 | -14 | 13.63  0.001 | HC<MSA |
| R superior temporal | 97 | 58 | 4 | -8 | 16.17  0.0004 | HC<MSA |
| R anterior cingulate | 203 | 2 | 44 | -4 | 15.46  0.0006 | HC<MSA |
| L middle temporal | 54 | -54 | -32 | -6 | 15.09  0.0006 | HC<MSA |
| R middle temporal | 148 | 50 | -22 | -4 | 16.44  0.0004 | HC<MSA |
| L middle temporal gyrus | 69 | -60 | -52 | -2 | 16.76  0.0004 | HC<MSA |
| L insula | 130 | -36 | -2 | 16 | 18.63  0.0002 | HC<MSA |
| Rolandic operculum | 388 | 54 | 14 | 12 | 17.63  0.0003 | HC<MSA |
| R inferior frontal gyrus | 36 | 42 | 38 | 12 | 15.69  0.0005 | HC<MSA |
| L inferior frontal gyrus | 62 | -40 | 34 | 16 | 14.90  0.0007 | HC<MSA |
| L superior frontal gyrus | 85 | -20 | 56 | 18 | 12.75  0.001 | HC<MSA |
| R superior temporal gyrus | 77 | 54 | -62 | 22 | 20.56  0.0001 | HC<MSA |
| R superior frontal gyrus | 406 | 18 | 56 | 28 | 20.42  0.0001 | HC<MSA |
| L parietal – supramarginal | 123 | -64 | -52 | 32 | 17.02  0.0003 | HC<MSA |
| R anterior cingulate | 50 | 6 | 30 | 32 | 11.15  0.002 | HC<MSA |
| R middle frontal | 109 | 32 | 30 | 42 | 20.60  0.0001 | HC<MSA |
| L middle frontal | 46 | -42 | 20 | 32 | 10.58  0.003 | HC<MSA |
| L supero-medial frontal | 30 | -8 | 48 | 46 | 13.23  0.001 | HC<MSA |
| L postcentral | 36 | -52 | -24 | 50 | 15.51  0.0005 | HC<MSA |
| R superior frontal | 98 | 22 | 20 | 58 | 23.16  5.51*10-5 | HC<MSA |
| L superior frontal | 96 | -20 | 12 | 62 | 12.58  0.001 | HC<MSA |

**Supplementary Table 2**: Regions of statistically significant effects in the GM obtained from the LME analysis that does not include the effect of atrophy (model 2). The statistical threshold is p<0.05 FDR corrected. The minimum cluster size is equal to 30 voxels. The number of voxels in the cluster are reported along with the MNI coordinate, the F- and p-values of the statistical peak of the difference.

**Analysis of the WM considering atrophy as a fixed effect in the LME model (model 1).**

| **MT** | | | | | | | | | |
| --- | --- | --- | --- | --- | --- | --- | --- | --- | --- |
| **Region** | | | **# of voxels** | | **x** | **y** | **z** | **F_peak_**  **P_Peak_** | **Comparison direction** |
| Brainstem | | | 567 | | 4 | -22 | -36 | 50.55  1.88*10-7 | HC>MSA |
| R frontal | | | 92 | | 50 | 2 | 26 | 28.78  1.45*10-5 | HC>MSA |
| **R1** | | | | | | | | | |
| No clusters | | | | | | | | | |
| **R2*** | | | | | | | | | |
| No clusters | | | | | | | | | |
| **T1ρ** | | | | | | | | | |
| brainstem | 695 | -2 | | -30 | | -28 | | 44.72  5.23*10-7 | HC<MSA |
| **T2ρ** | | | | | | | | | |
| brainstem | 300 | -4 | | -30 | | -18 | | 104.31  2.09*10-10 | HC<MSA |

**Supplementary Table 3**: Regions of statistically significant effects in the WM obtained from the LME analysis that considers the effect of atrophy (model 1). The statistical threshold is p<0.05 FDR corrected. The minimum cluster size is equal to 30 voxels. The number of voxels in the cluster are reported along with the MNI coordinate, the F- and p-values of the statistical peak of the difference.

**Analysis of the WM without considering tissue volume as a fixed effect in the LME model (model 2).**

| **MT** | | | | | | | |
| --- | --- | --- | --- | --- | --- | --- | --- |
| **Region** | **# of voxels** | **x** | | **y** | **z** | **F_peak_**  **P_Peak_** | **Comparison direction** |
| Pons | 1351 | 6 | | -22 | -38 | 82.52  1.51*10-9 | HC>MSA |
| R sub-lobar | 106 | 20 | | 10 | 10 | 16.68  0.0004 | HC>MSA |
| R frontal | 272 | 50 | | 2 | 26 | 26.76  1.13*10-5 | HC>MSA |
| **R1** | | | | | | | |
| Pons | 1222 | -18 | | -36 | -38 | 52.25  1.12*10-7 | HC>MSA |
| R parahippocampal | 84 | 24 | | -34 | -8 | 14.29  0.0008 | HC>MSA |
| R sub-lobar | 422 | 8 | | 0 | 2 | 20.77  0.0001 | HC>MSA |
| L sub-lobar | 148 | -8 | | 0 | 4 | 17.12  0.0003 | HC>MSA |
| Corpus callosum | 330 | -6 | | -26 | 24 | 16.34  0.0004 | HC>MSA |
| R frontal | 175 | 40 | | -8 | 36 | 13.36  0.001 | HC>MSA |
| **R2*** | | | | | | | |
| L Brainstem | 308 | | -10 | -26 | -24 | 35.04  3.06*10-6 | HC>MSA |
| L temporal | 44 | | -38 | -38 | 0 | 24.76  3.58*10-5 | HC>MSA |
| **T1ρ** | | | | | | | |
| Brainstem | 1662 | -10 | | -40 | -34 | 63.30  1.96*10-8 | HC<MSA |
| R temporal lobe | 65 | 40 | | -28 | -8 | 16.65  0.0004 | HC<MSA |
| **T2ρ** | | | | | | | |
| Brainstem | 876 | -4 | | -30 | -18 | 107.54  10*10-11 | HC<MSA |
| R anterior cerebellum | 211 | 18 | | -42 | -28 | 37.85  1.66*10-6 | HC<MSA |
| R temporal | 90 | 42 | | -22 | -2 | 18.64  0.0002 | HC<MSA |

**Supplementary Table 4**: Regions of statistically significant effects in the WM obtained from the LME analysis that does not consider the effect of atrophy (model 2). The statistical threshold is p<0.05 FDR corrected. The minimum cluster size is equal to 30 voxels. The number of voxels in the cluster are reported along with the MNI coordinate, the F- and p-values of the statistical peak of the difference.

**Bibliography**

1. Draganski B, Ashburner J, Hutton C, Kherif F, Frackowiak RSJ, Helms G, et al. Regional specificity of MRI contrast parameter changes in normal ageing revealed by voxel-based quantification (VBQ). NeuroImage. 2011;55:1423–34.

2. Michaeli S, Grohn H, Sorce DJ, Kauppinen R, Springer Jr. CS, Uğurbil K, et al. Exchange‐influenced T2ρ contrast in human brain images measured with adiabatic radio frequency pulses. Magn Reson Med [Internet]. 2005 [cited 2024 May 27]; Available from: https://onlinelibrary.wiley.com/doi/10.1002/mrm.20428

3. Michaeli S, Sorce DJ, Springer CS, Ugurbil K, Garwood M. T1rho MRI contrast in the human brain: modulation of the longitudinal rotating frame relaxation shutter-speed during an adiabatic RF pulse. J Magn Reson San Diego Calif 1997. 2006;181:135–47.

4. Michaeli S, Sorce DJ, Idiyatullin D, Ugurbil K, Garwood M. Transverse relaxation in the rotating frame induced by chemical exchange. J Magn Reson. 2004;169:293–9.

5. Helms G, Dathe H, Kallenberg K, Dechent P. High-resolution maps of magnetization transfer with inherent correction for RF inhomogeneity and T1 relaxation obtained from 3D FLASH MRI. Magn Reson Med. 2008;60:1396–407.

6. Weiskopf N, Lutti A, Helms G, Novak M, Ashburner J, Hutton C. Unified segmentation based correction of R1 brain maps for RF transmit field inhomogeneities (UNICORT). NeuroImage. 2011;54:2116–24.

7. Weiskopf N, Callaghan MF, Josephs O, Lutti A, Mohammadi S. Estimating the apparent transverse relaxation time (R2(*)) from images with different contrasts (ESTATICS) reduces motion artifacts. Front Neurosci. 2014;8:278.

8. Ashburner J. A fast diffeomorphic image registration algorithm. NeuroImage. 2007;38:95–113.

9. Good CD, Johnsrude IS, Ashburner J, Henson RN, Friston KJ, Frackowiak RS. A voxel-based morphometric study of ageing in 465 normal adult human brains. NeuroImage. 2001;14:21–36.

10. Callaghan MF, Freund P, Draganski B, Anderson E, Cappelletti M, Chowdhury R, et al. Widespread age-related differences in the human brain microstructure revealed by quantitative magnetic resonance imaging. Neurobiol Aging. 2014;35:1862–72.
